# Ring finger protein 19A is overexpressed in non‐small cell lung cancer and mediates p53 ubiquitin‐degradation to promote cancer growth

**DOI:** 10.1111/jcmm.16674

**Published:** 2021-06-29

**Authors:** Yu Cheng, Yujiao Hu, Huanxi Wang, Zhi Zhao, Xizi Jiang, Yao Zhang, Jiameng Zhang, Yue Tong, Xueshan Qiu

**Affiliations:** ^1^ Department of Pathology College of Basic Medical Sciences and First Affiliated Hospital China Medical University Shenyang China; ^2^ Department of Pathology Cancer Research Laboratory Chengde Medical College Chengde China; ^3^ Department of Pathology Zhengzhou Yihe Hospital Affiliated to Henan University Zhengzhou China

**Keywords:** carcinogenesis, non‐small cell lung cancer, p53, RNF19A, ubiquitin

## Abstract

The expression pattern, biological functions and the related mechanisms of the ring finger protein 19A (RNF19A) in non‐small cell lung cancer (NSCLC) remain poorly understood. This study aimed to explore the role of RNF19A, as well as the underlying potential mechanism, in the development of NSCLC. Here, we found that RNF19A was overexpressed in NSCLC tissues, and RNF19A expression in NSCLC tissue samples was associated with NSCLC carcinogenesis and poor outcome. RNF19A promoted the proliferation of NSCLC cells and inhibited apoptosis. RNF19A reduced p53, p21 and BAX expression and induced Cyclin D1, CDK4, CDK6 and BCL2 expression. The inhibitory effect of *RNF19A* knockdown on proliferation was partially rescued by *p53* silencing. RNF19A interacted with p53, shortened p53 half‐life and mediated p53 ubiquitin‐degradation. Collectively, we suggest that RNF19A plays a critical oncogenic role in lung carcinogenesis by disrupting the function of p53. RNF19A may serve as a new biomarker and/or target for NSCLC management.

## 1 INTRODUCTION

Lung cancer is the most frequent cause of cancer‐related deaths worldwide, with approximately 2.09 million new cases and 1.76 million deaths every year.^1^ Most (approximately 85%) lung carcinoma cases are non‐small cell lung cancers (NSCLCs) at initial presentation. Histologically, subtypes of NSCLC are adenocarcinoma (38.5%), squamous cell carcinoma (20%) and large cell carcinoma (3%).^2^ Despite the latest advancements in new treatment modalities, including surgery, chemotherapeutic agents and radiotherapy, the overall 5 year survival of patients with NSCLC is less than 20%.^3^ Meanwhile, the potential molecular mechanism of NSCLC is unknown and greater efforts should be directed towards the development of anti‐NSCLC strategies.

The RING‐in‐between‐RING (RBR) E3 ubiquitin ligases (RBR E3 ligases) are recently categorized E3 members, which contain a highly conserved catalytic unit consisting of a RING1, an in‐between RING (IBR) and a RING2 domain.^4^, ^5^ Contrary to classical RING and HECT E3 ligases, RBR E3 ligases catalyse ubiquitin transfer to a substrate through a combined RING/HECT‐like mechanism, in which the RING1 domain facilitates E2 discharge to directly form a thioester intermediate with a cysteine in RING2.^6^ These E3 ligases are involved in important cellular events including cellular and stress signalling, regulation of post‐translational modifications and protein stability, and cycle control.^7^ Misregulation of RBR proteins often leads to diverse diseases, including various cancers. For instance, RBR E3 ligases, including Parkin,^8^ RNF144A,^9^, ^10^ RBCK1^11^, ^12^ and RNF216^13^ are involved in lung, breast and colorectal cancers through ubiquitin‐mediated degradation, indicating the vital function of RBR proteins in carcinogenesis.

Ring finger protein 19A (RNF19A), also known as Dorfin, is a poorly understood member of RBR E3 ligases, which carries three highly conserved domains including two RING finger motifs and an IBR motif at its N terminus.^14^ The biological functions and action mechanisms of RNF19A remain largely unknown. Available literature confirms the independent function of RNF19A by controlling protein quality, and its potential involvement in the development of neurodegenerative diseases.^15^, ^16^, ^17^, ^18^ It was reported that the mRNA levels of *RNF19A* increases in the serum of patients suffering from prostate cancer.^19^ However, to date, there are no studies on the intracellular RNF19A expression pattern, as well as its direct role and mechanism, in cancers. Here, we aimed to explore the biological functions, clinical application and underlying molecular mechanisms of RNF19A in NSCLC.

## 2 MATERIALS AND METHODS

### 2.1 Human samples

NSCLC specimens from 136 patients, who underwent curative surgery in the First Affiliated Hospital of China Medical University in Shenyang, China, were obtained from 2014 to 2016. None of the patients had received pre‐operative chemoradiotherapy. Besides, we collected 30 normal paracancer tissues (control samples) from these patients. Eight NSCLC specimens and matching normal paracancer tissues were rapidly placed in liquid nitrogen within 10 minutes and then stored at −80°C in an ultra‐low temperature refrigerator until Western blotting was performed. All patients provided written informed consent, and all experiments were approved by the Medical Research Ethics Committee of China Medical University.

### 2.2 Bioinformatic analyses

Differences in the transcriptional expression of *RNF19A* between NSCLC tissues and their corresponding non‐tumour samples were evaluated based on the data obtained from the Oncomine database (http://www.oncomine.org) ^20^ The conditions were set as follows: Data type, mRNA; *P‐*value < 0.01; fold change >1.5; gene rank, all. The prognostic value of *RNF19A* in NSCLC was analysed via Gene Expression Profiling Interactive Analysis (GEPIA) (http://gepia.cancer‐pku.cn/) ^21^ The median gene expression level, 95% confidence intervals (CIs), HRs and *P* values were retrieved from the GEPIA database. A *P‐*value < 0.05 was considered to denote statistically significant results.

### 2.3 Immunohistochemistry (IHC)

The resected specimens were fixed using a 10% formaldehyde solution, embedded in paraffin and cut into 4 μm thick serial sections. After incubating at 70℃ for 2 hours, the sections were dewaxed and rehydrated in xylene and graded alcohol. A citrate solution (0.01 mol/L) (Maixin‐Bio) was used for antigen retrieval under high temperature and pressure for 3 minutes. The activity of endogenous peroxidase was blocked via incubation with 0.3% H_2_O_2_ for 20 minutes, and then, each section was blocked in 5% goat serum for 30 minutes.

All the sections were incubated with a drop of anti‐RNF19A rabbit polyclonal antibody (1:50 in 2% BSA; #PA5‐54861; Invitrogen, Carlsbad, CA, USA) overnight at 4°C. After soaking in 1 × PBS thrice for 5 minutes, the sections were incubated with the substrate provided in the Elivision^TM^super HRP (Mouse/Rabbit) IHC Kit (KIT9921; MaiXin) according to the manufacturer's instructions. Next, 3,3'‐diaminobenzidine (DAB) staining and haematoxylin counterstaining were performed. Finally, the sections were dehydrated using gradient ethanol, cleared by dimethylbenzene and mounted with a neutral gum seal tablet. Immunohistochemical staining results were evaluated by two experienced pathologists in a double‐blinded manner. The scoring system for positively stained cancer cells was established as described previously.^22^ Briefly, based on the staining intensity, cells characterized by no staining, weak staining, moderate staining and strong staining were scored as 0, 1, 2 and 3, respectively. According to the range of positive cell distribution, tissues characterized by 0%, 1‐30%, 31‐70% and 71‐100% positive cells were scored as 0, 1, 2 and 3, respectively. The two scoring results were multiplied to give a total score ranging from 0 to 9. Tissues with total scores of 0‐3 were considered to show low expression of RNF19A, and those with scores of 3‐9 were considered to show high expression of RNF19A.

### 2.4 Cell culture, transfection and transduction

Human non‐small cell lung carcinoma cell lines A549, H292, H460, H661, H1299 and SK‐MES‐1 were purchased from the Cell Bank of the China Academy of Sciences (Shanghai, China). Human bronchial epithelial cells (HBE) were obtained from the ATCC. According to the ATCC protocol, HBE cells were grown in DMEM, while A549, H292, H460, H661 and H1299 cells were grown in RPMI 1640 medium, and SK‐MES‐1 cells were cultured in MEM. Ten per cent foetal bovine serum was added to all media, and all cells were cultured at 37°C in a 5% CO_2_‐containing atmosphere. In vitro transfection of two small interfering RNAs (siRNAs) and *RNF19A* expression plasmids were performed using Lipofectamine 3000 reagent (Invitrogen) following the manufacturer's protocol. Cells were transfected with siRNA targeting *RNF19A* and scrambled control siRNA (Ribobio, Guangzhou, China) for 72 hours. The siRNA sequence against *RNF19A* was as follows: siRNF19A‐1, 5'‐GATCCATTCTGAATTCCTA‐3'; siRNF19A‐2, 5'‐GCAAGTAGATATTGAGTCA‐3'. An *RNF19A* DNA fragment was cloned into the PCMV3 vector, containing a FLAG sequence, which was obtained from Sino Biological.

### 2.5 MTT and colony formation assays

The viability of lung cancer cells was analysed using the MTT assay. Briefly, the treated A549, H460 and H1299 cells were seeded in 96 well microplates at a density of 3000, 1500 and 2000 cells per well, respectively. The cells were incubated with a 10% MTT solution (100 µl/well) for 3.5 hours, and the absorbance was measured at 490 nm. Measurements were taken once per day for five consecutive days. For the colony formation assay, approximately 500 cells/well were plated into a six‐well plate and incubated for 8‐12 days. The colonies were fixed with cold methanol for 15 minutes, stained with 0.5% crystal violet solution for 30 minutes, and finally, colonies consisting of more than 50 cells were counted using ImageJ.

### 2.6 Apoptosis analysis

Cell apoptosis assay was performed via Annexin V‐FITC/PI staining according to the manufacturer's instructions (Annexin V‐FITC Apoptosis Detection Kit, #KGA105‐KGA108, KeyGene BioTech, Nangjing, China). Briefly, the transfected cells were incubated for 48 hours, collected using 0.25% trypsin without EDTA and washed twice with pre‐cooled PBS. Cell pellets were suspended in 500 μl binding buffer and then stained with 5 μl Annexin V‐FITC and 5 μl propidium iodide. After letting the reaction happen at room temperature and away from light for 5‐15 minutes, the stained cells were detected using flow cytometry.

### 2.7 Western blotting

Frozen tissues and cultured cells were harvested and lysed with NP‐40 lysis solution containing protease inhibitors. After measuring the protein concentration of the mixed lysates, 40 μg of supernatant protein was separated via SDS‐PAGE (12% gels) and subsequently transferred onto PVDF membranes. Then, the membranes were blocked with 5% skimmed milk for 2 hours at about 37°C and incubated with appropriate primary antibodies overnight at 4°C. After washing thrice with TBST, the membranes were incubated with freshly prepared HRP‐conjugated anti‐mouse (#SA00001‐1)/ rabbit (#SA00001‐2) secondary antibody (1:10 000; Proteintech, Wuhan, China) at 37°C for 1 h. Hybridization signals were detected via ECL detection reagents using a Bio‐Imaging System (UVP Inc, Upland). An anti‐RNF19A polyclonal antibody (#PA5‐54861) was obtained from Invitrogen; an anti‐CDK4 (#12790) antibody was purchased from Cell Signaling Technology (Beverly, MA, USA). Finally, anti‐p53 (#10442‐1‐AP), anti‐p21 (#10355‐1‐AP), anti‐Cyclin D1 (#60186‐1‐Ig), anti‐CDK6 (#14052‐1‐AP), anti‐BAX (#60267‐1‐Ig), anti‐BCL2 (#12789‐1‐AP) and anti‐GAPDH (#60004‐1‐Ig) antibodies were obtained from Proteintech. All primary antibodies were diluted 1:1000, except for GAPDH which was used at 1:10 000.

### 2.8 Quantitative real‐time PCR (qRT‐PCR)

Total RNA was extracted using TRIzol reagent (TransGen Biotech, Beijing, China), and the cDNA was reverse transcribed using the FastQuant RT Kit (TIANGEN Biotech, Beijing, China) following the manufacturer's instructions. qRT‐PCR was performed using a 7900HT fast real‐time PCR system (Applied Biosystems, Foster City, CA, USA). The reaction conditions for qRT‐PCR were as follows: Pre‐degeneration at 95°C for 15 minutes; followed by 40 cycles of denaturation at 95°C for 10 seconds and annealing at 60°C for 32 seconds. *GAPDH* was used as an internal control, and relative gene expression was calculated using the 2^−ΔΔCt^ method.

Sequences of designed primers were as follows: For *RNF19A*, forward 5'‐AGCATAGGGGAGGGAAGTGT‐3' and reverse 5'‐TACCATGGCACTTCCTGACA‐3'; for *p53*, forward 5'‐ CAGCACATGACGGAGGTTGT‐3' and reverse 5'‐ TCATCCAAATACTCCACACGC‐3'; for *GAPDH*, forward 5'‐CGGAGTCAACGGATTTGGTCGTAT‐3' and reverse 5'‐AGCCTTCTCCATGGTGGTGAAGAC‐3'.

### 2.9 Protein half‐life detection

After being transfected with 75 nmol/L siRNF19A or siControl for 24 hours, A549 and H460 cells were treated with 3 μg/ml cycloheximide (CHX) and collected after 0, 0.5, 1, 1.5, 2 and 2.5 hours of treatment. Protein was extracted for SDS‐PAGE and Western blotting using anti‐RNF19A or anti‐p53 antibody. GAPDH served as an internal reference and the half‐life of p53 was estimated.

### 2.10 Co‐immunoprecipitation and ubiquitination assays

Co‐immunoprecipitation and ubiquitination assays were carried out as described previously.^23^ Briefly, the cell lysates were blocked with 40 μl Protein A + G agarose beads (Beyotime Biosciences) at 4°C for 2 hours and immunoprecipitated with 4‐10 μg mouse primary anti‐p53 (#60283‐2‐Ig, Proteintech), mouse primary anti‐FLAG (#HT201‐01, TransGen Biotech) or rabbit primary anti‐p53 (#10442‐1‐AP, Proteintech) antibodies overnight at 4°C. The immune complexes were captured by 30 μl Protein A + G agarose beads and visualized via Western blotting.

For the ubiquitination assay, A549 cells were transfected with specified siRNA or plasmids for 48 hours and then treated with MG132 (MedChemExpress, Monmouth Junction) for 24 hours before they were collected and lysed.

After centrifuging, the supernatant was immunoprecipitated using an anti‐p53 (1:1000, #10442‐1‐AP/#60283‐2‐Ig, Proteintech) antibody overnight at 4°C. Samples were then incubated with Protein A + G agarose (Beyotime Biosciences) and washed there times in NP‐40 lysis buffer. Agarose and 20 μg of the protein immunocomplex were immunoblotted using an anti‐HA antibody (1:1000, #51064‐2‐AP, Proteintech).

### 2.11 Statistical analysis

The SPSS 19.0 statistical software (SPSS Inc) and the GraphPad Prism 7.0 software (GraphPad Software, Inc) were used to analyse the experimental data. The correlation between RNF19A expression and clinicopathological characteristics was examined using the chi‐squared test. Data were presented as the mean ± standard error (SE), and quantitative analysis was performed using Student's *t* test if not stated otherwise. The level of significance was set at *P < .05, ** *P* < .01 and ***P < .001.

## 3 RESULTS

### 3.1 RNF19A is highly expressed in NSCLC and is associated with poor patient outcome

**FIGURE 1:**
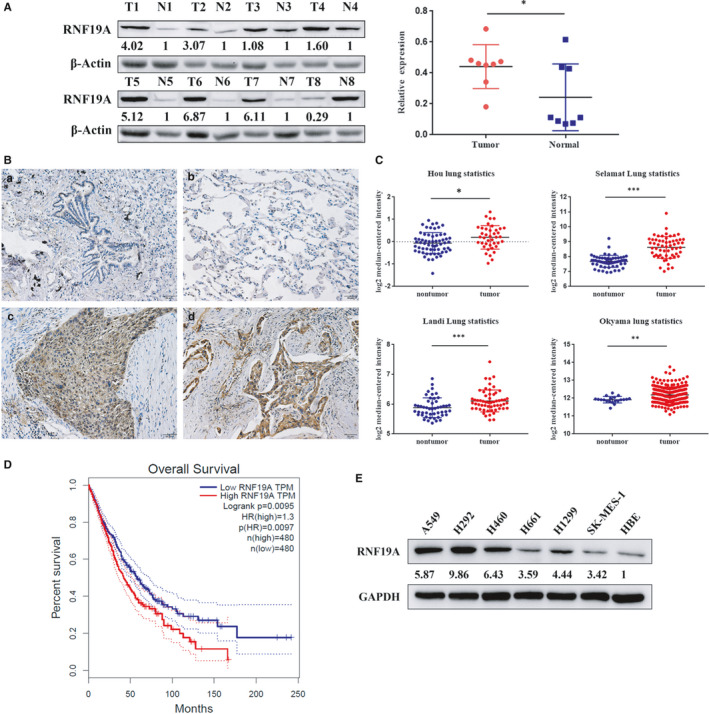
RNF19A is highly expressed in NSCLC tissues and correlates with poor prognosis. A, Expression of RNF19A in eight matching NSCLC and non‐tumour tissues was detected via Western blotting. T, tumour tissue; N, non‐tumour tissue. *P < .05. B, IHC staining representative images of RNF19A in NSCLC and non‐tumour specimens. a, bronchiole tissue; b, alveolar tissue; c, squamous cell carcinoma tissue; d, adenocarcinoma tissue. C, *RNF19A* mRNA expression comparison between NSCLC and non‐tumour tissues from the Oncomine database. *P < .05, **P < .01 and ***P < .001. D, RNF19A expression correlates with poor prognosis in patients with NSCLC, obtained from GEPIA data sets. *P < .05, ** *P* < .01 and ***P < .001. E, Immunoblot of RNF19A confirms high expression of RNF19A in NSCLC cell lines. IHC, immunohistochemistry; NSCLC, non‐small cell lung cancer

To explore the RNF19A expression pattern in NSCLC, we examined the expression of RNF19A in matching cancerous and normal tissues from eight patients with NSCLC via Western blotting. RNF19A expression levels were notably increased in six out of eight NSCLC tissues compared with those in the normal adjacent tissues (Figure 1A *P* < .05). Meanwhile, IHC was carried out in 136 NSCLC specimens and 30 normal lung tissues to examine the expression of RNF19A in patients with NSCLC who underwent curative surgery. Our data showed that RNF19A expression was significantly higher in NSCLC tissues (detection rate: 79.4%) than that in normal tissues (detection rate: 30.0%, Table 1, Figure 1B *P* < .01). The chi‐squared test indicated that high RNF19A expression was notably associated with tumour size (*P* < .05) and TNM stage (*P* < .05) of patients with NSCLC (Table 2). However, no significant association between RNF19A expression and other clinical features, such as histological type or differentiation degree, was observed.

**TABLE 1:** RNF19A expression in normal lung and NSCLC tissues

Group	n	RNF19A		*χ^2^*‐value	*P*‐value
		−	+		
Normal	30	21	9	28.843	.000
NSCLC	136	28	108		

Abbreviations: NSCLC, Non‐small cell lung cancer.

**TABLE 2:** Correlation between RNF19A protein expression and clinicopathologic variables of patients with NSCLC

Clinicopathological characteristics	n	RNF19A		*χ^2^*‐value	*P*‐value
		−	+		
Gender					
Male	89	15	74	2.197	.138
Female	47	13	34		
Age (y)					
≤60	68	14	54	0.000	1.000
>60	68	14	54		
Histological type					
Squamous cell carcinoma	69	12	57	0.876	.349
Adenocarcinoma	67	16	51		
Differentiation degree					
Well‐moderate	81	19	62	1.008	.315
Poor	55	9	46		
Tumour size					
≤3 cm	66	19	47	5.273	.022
>3 cm	70	9	61		
lymph node metastasis					
Negative	90	22	68	2.420	.120
Positive	46	6	40		
TNM stage					
Ⅰ + Ⅱ A	73	20	53	4.469	.035
Ⅱ B + Ⅲ	63	8	55		

Abbreviations: NSCLC, Non‐small cell lung cancer.

Consistently, bioinformatics analysis of data sets retrieved from the Oncomine database also demonstrated a significantly higher *RNF19A* expression in lung cancer tissues compared with normal lung tissues (Figure 1C  < .05). Moreover, the analysis of lung cancer survival data from the GEPIA database suggested that patients with NSCLC with higher *RNF19A* expression showed shorter overall survival (Figure 1D  < .05). These results strongly suggested that high RNF19A expression might play a critical role during NSCLC progression.

RNF19A expression was investigated in NSCLC cell lines, A549, H292, H460, H661, H1299 and SK‐MES‐1 using Western blotting. Compared with those in HBE cells, RNF19A levels were mostly elevated in tumour cell lines (Figure 1E). A549 and H460 cells were used for the subsequent experiments because of their moderate RNF19A expression.

### 3.2 RNF19A promotes NSCLC cell growth

**FIGURE 2:**
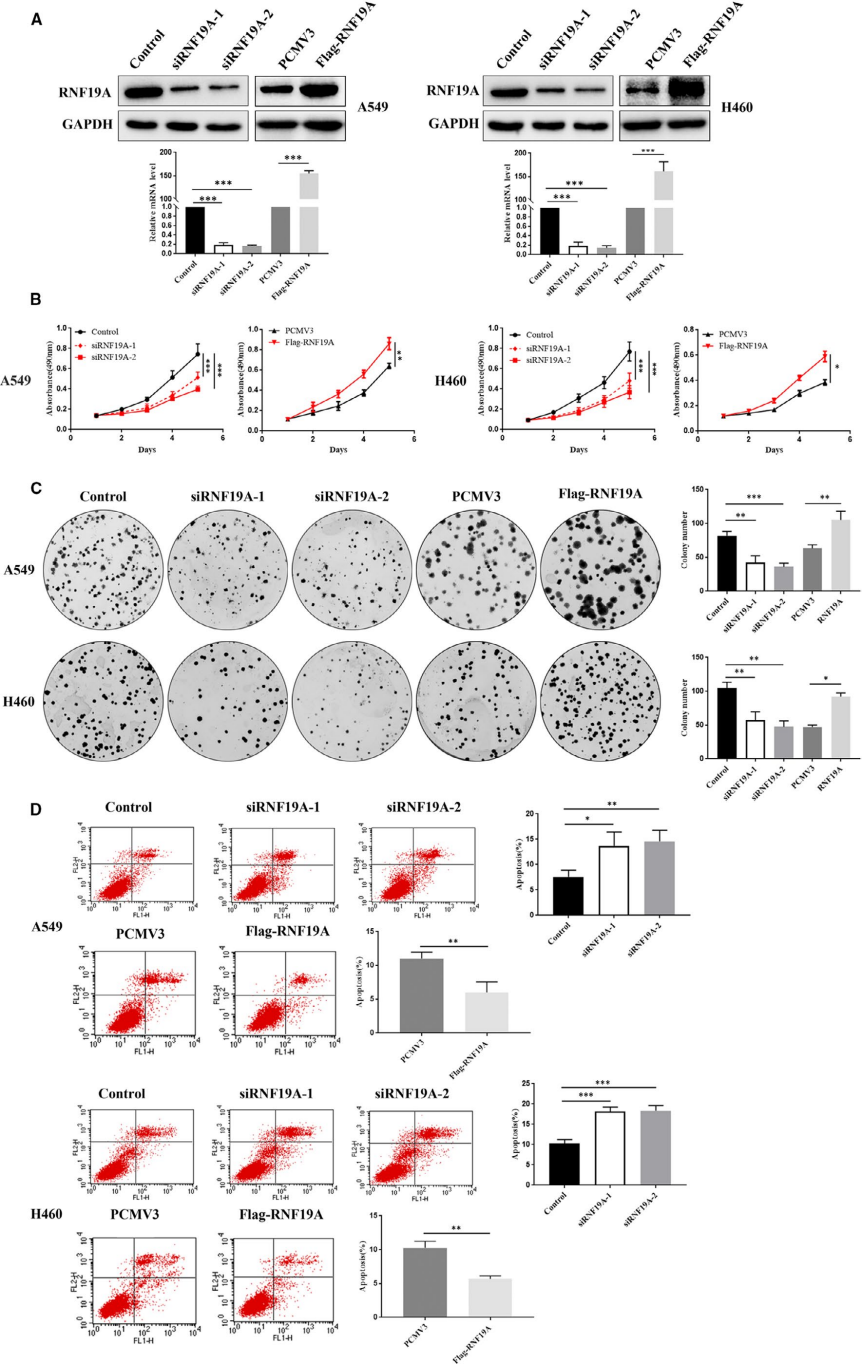
RNF19A promotes NSCLC cell proliferation and inhibits apoptosis. A, RNF19A knockdown and overexpression was detected via Western blotting and qRT‐PCR. B, Viability of transfected A549 and H460 cells was measured using the MTT assay. C, Colony formation was detected in transfected A549 and H460 cells. D, Transfected A549 and H460 cells were stained with Annexin V‐FITC/PI. Stained cells were analysed via flow cytometry. *P < .05, ** *P* < .01 and ***P < .001. Data are presented as the mean ± SE of three independent experiments. SE, standard error

To further explore the possible biological function of RNF19A in NSCLC, we used RNF19A‐specific siRNA or a PCMV3‐RNF19A vector to suppress or enhance RNF19A expression in A549 and H460 cell lines, respectively. qRT‐PCR and Western blotting were performed to identify the transfection efficiency. The results showed that RNF19A siRNA effectively blocked RNF19A expression at the protein and mRNA level, while RNF19A was overexpressed in PCMV3‐RNF19A vector‐transfected cells (Figure 2A). MTT analysis showed that *RNF19A* knockdown suppressed the growth of A549 and H460 cells. On the contrary, *RNF19A* overexpression promoted growth (Figure 2B  < .05). Moreover, *RNF19A* knockdown inhibited the colony formation in A549 and H460 cells, while *RNF19A* overexpression dramatically increased this capacity (Figure 2C  < .05). Furthermore, *RNF19A* knockdown promoted the apoptosis of A549 and H460 cells, yet, *RNF19A* overexpression had opposite effects (Figure 2D  < .05). These results suggested that *RNF19A* might act as an oncogene in NSCLC.

### 3.3 RNF19A represses p53 expression and regulates p53 downstream signalling

**FIGURE 3:**
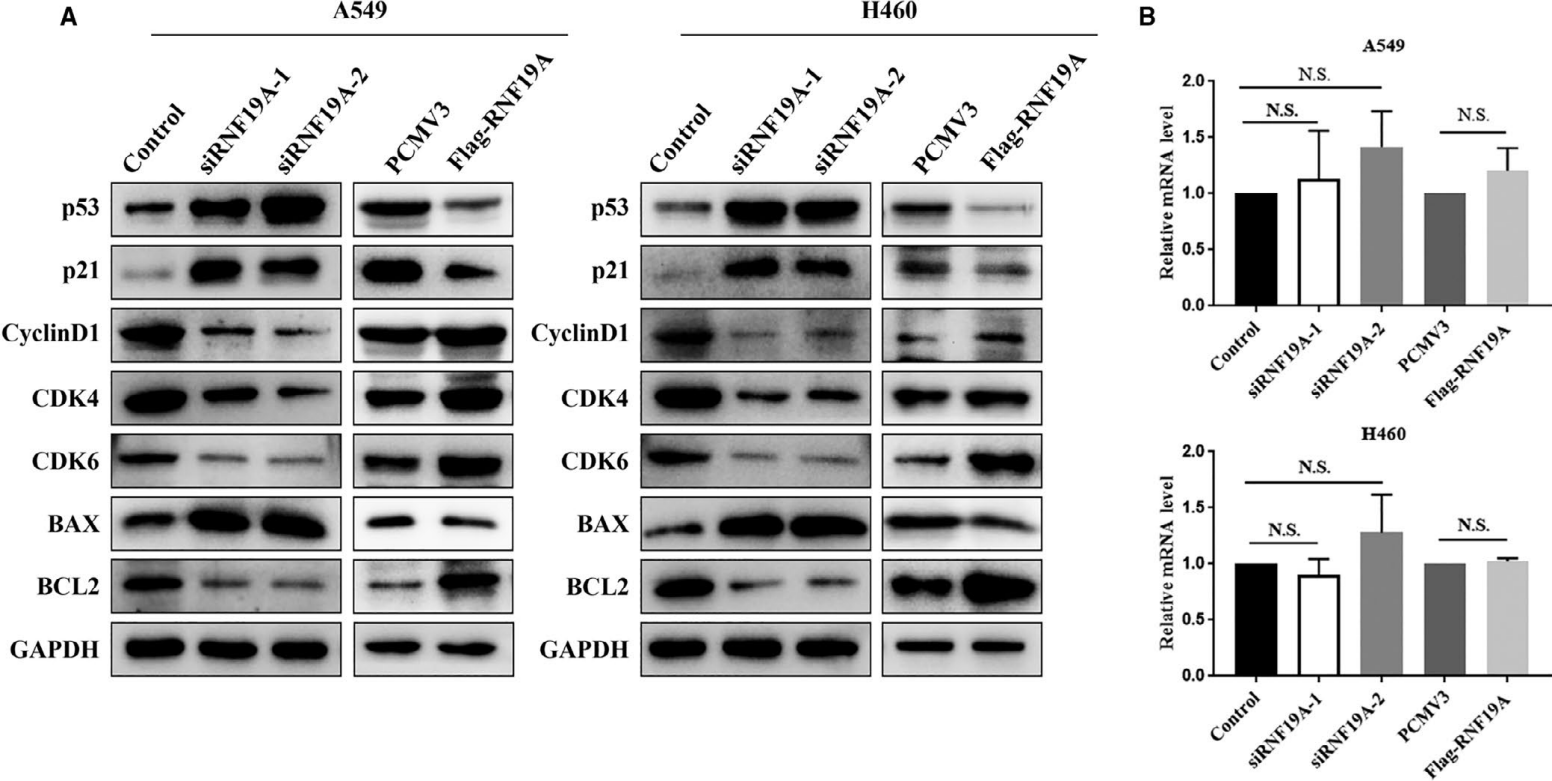
RNF19A represses the expression of p53 and regulates p53 downstream molecules. A, Expression of p53 and its downstream molecules was evaluated via Western blotting in A549 and H460 cells transfected with RNF19A siRNA and RNF19A overexpression plasmid. B, *TP53* mRNA expression in the transfected cells was evaluated using qRT‐PCR. Data are presented as the mean ± SE of three independent experiments. N.S., not significant; SE, standard error

Our results showed that RNF19A supported the growth of NSCLC cells. We further explored the possible mechanisms of RNF19A‐prompted proliferation and apoptosis inhibition in cancer cells and paid attention to the regulation of p53 by RNF19A. First, we detected p53 expression at protein and mRNA levels after transfection with RNF19A siRNA or a plasmid that carried *RNF19A*. Our data revealed that knockdown and overexpression of *RNF19A* significantly up‐regulated and down‐regulated p53 expression at the protein level, respectively (Figure 3A); however, the mRNA expression of *p53* did not change (Figure 3B). Next, we detected the effects of RNF19A on the expression levels of p53 downstream proteins. *RNF19A* knockdown significantly increased the expression levels of the cell cycle inhibitor p21 (CDKN1A) and the pro‐apoptotic factor BAX, and decreased the expression of Cyclin D1, CDK4, CDK6, as well as the pro‐survival factor BCL2, in A549 and H460 cells. Overexpression of *RNF19A* had the opposite effect (Figure 3A). These data suggested that RNF19A might inhibit the p53 signalling pathway in NSCLC cells.

### 3.4 RNF19A exerts tumour‐promoting effects through p53

**FIGURE 4:**
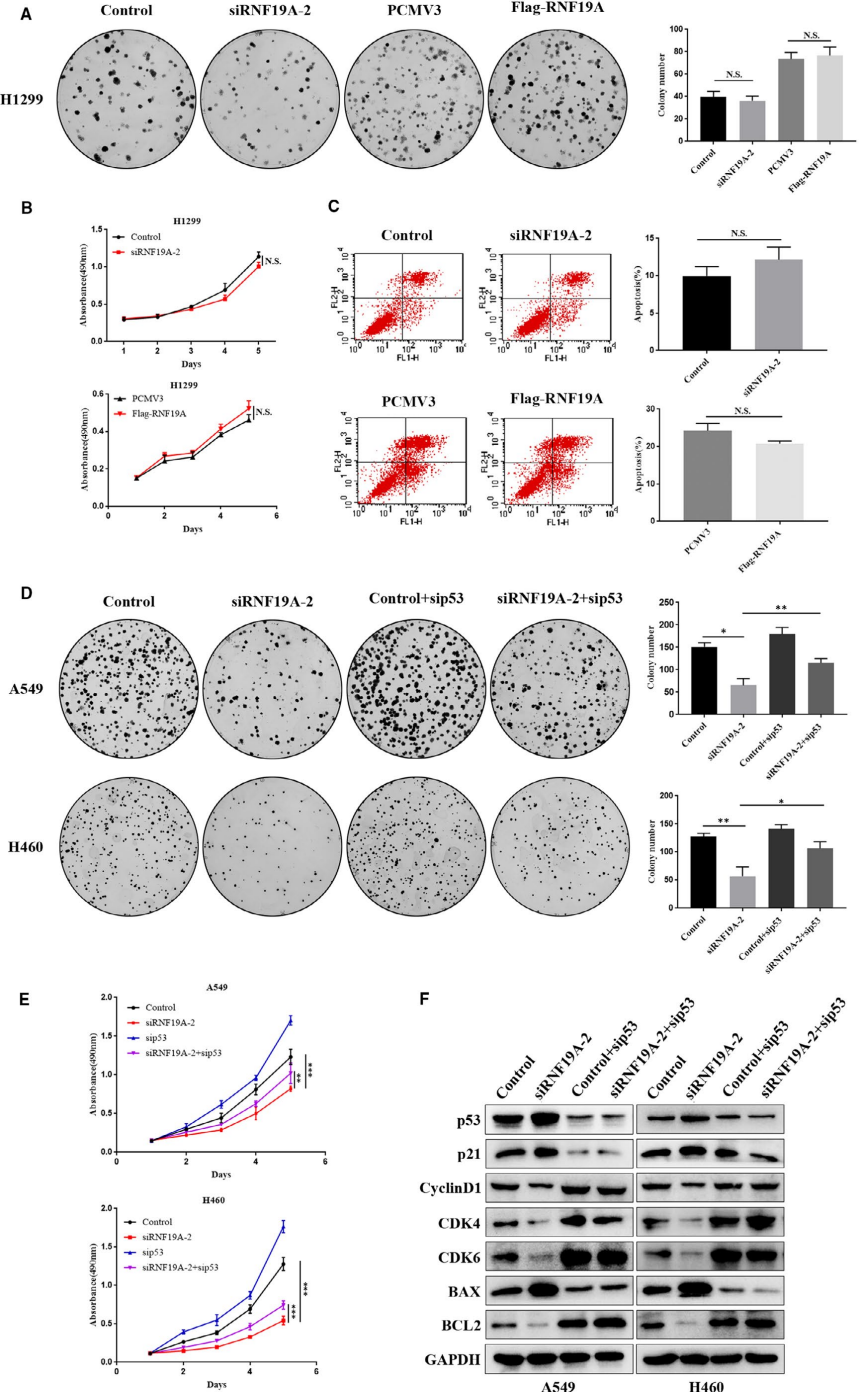
RNF19A exerts tumour‐promoting effects through p53. A‐C, Knockdown or overexpression of *RNF19A* had no notable effect on the colony formation, cell viability and apoptosis of p53‐null (H1299) cells. D, Knockdown of p53 significantly increased the reduction in the colony formation of RNF19A siRNA‐transfected A549 and H460 cells. E, p53 depletion attenuates the inhibitory effect of RNF19A knockdown on A549 and H460 cells. F, Changes in the expression of p53 and its downstream proteins were detected via Western blotting after transfection of RNF19A‐silenced cells with p53 siRNA. Statistical results are presented as the mean ± SE, N.S., not significant,*P < .05, ** *P* < .01, and ***P < .001. SE, standard error

The above results suggested that RNF19A promoted cellular proliferation and inhibited apoptosis in A549 and H460 cells, which endogenously express wild‐type *TP53*. However, *RNF19A* knockdown or overexpression did not affect the proliferation and apoptosis in *p53*‐null (H1299) or *p53*‐mutant (SK‐MES‐1) cells (Figure 4A‐C, Figure S1A‐C). This observation suggested that RNF19A plays a pro‐oncogenic through p53.

To further confirm whether p53 is essential for the oncogenic role of RNF19A, we carried out a rescue experiment by repressing *p53* expression in RNF19A siRNA‐transfected cells. As shown in Figure 4D‐E, p53 knockdown dramatically reduced the proliferation inhibition effect of *RNF19A* knockdown in A549 and H460 cells. In addition, *p53* knockdown reversed the effect of *RNF19A* knockdown on the expression of p53 downstream proteins (Figure 4F). These data suggested that RNF19A promoted NSCLC growth at least partially through p53.

### 3.5 RNF19A interacts with p53 to promote p53 ubiquitination

**FIGURE 5:**
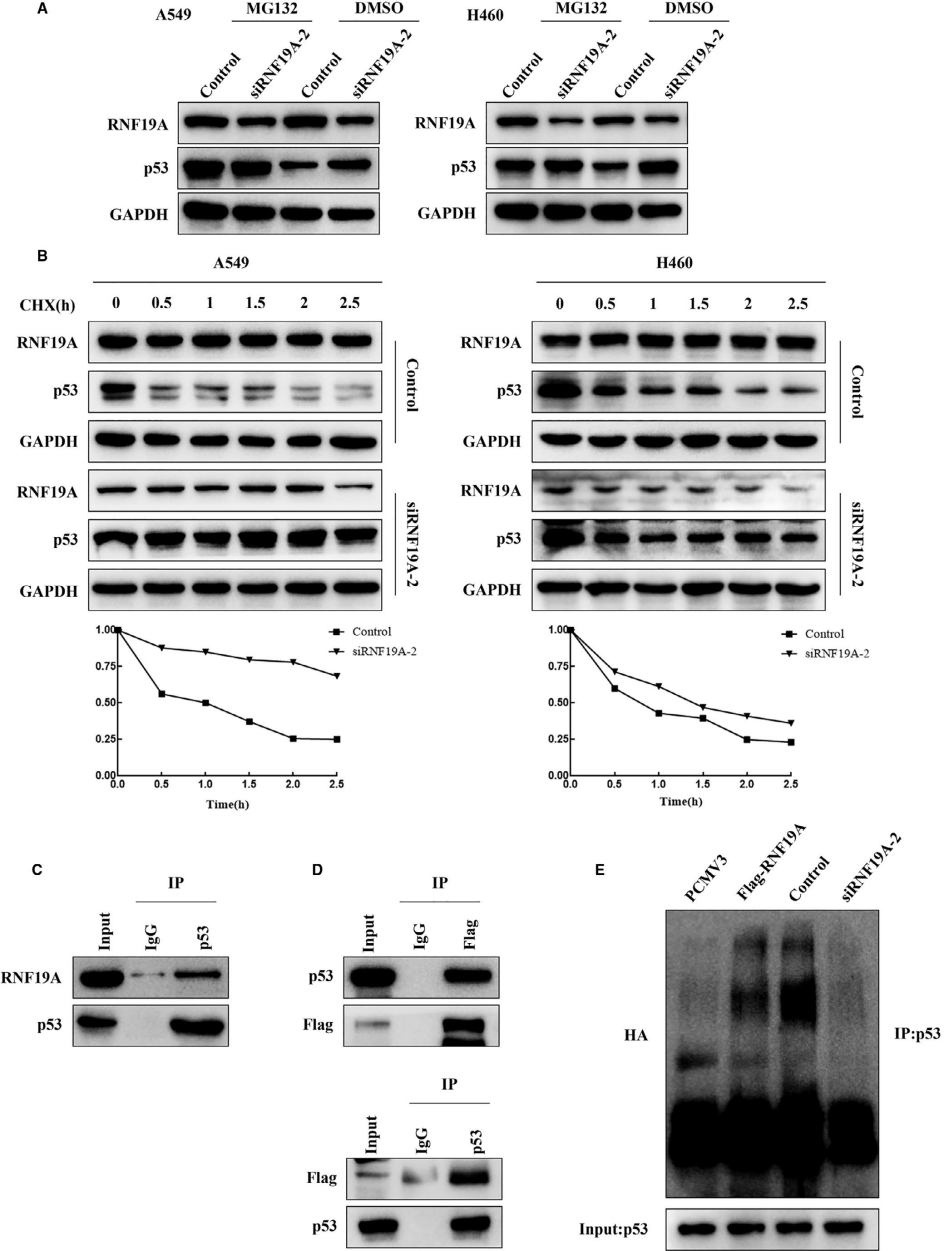
RNF19A interacts with p53 and promotes p53 ubiquitination. A, RNF19A‐silenced A549 and H460 cells were treated with MG132 and DMSO (control). RNF19A and p53 expression levels in the treated cells were detected via Western blotting. B, RNF19A‐silenced A549 and H460 cells were treated with CHX at the indicated time points. RNF19A and p53 expression levels in the treated cells were detected via Western blotting and the half‐life of p53 was calculated. C, Endogenous p53 and RNF19A form a protein complex in A549 cells. D, RNF19A co‐immunoprecipitates with p53 in A549 cells transfected with p53 and FLAG‐tagged RNF19A. E, A549 cells transfected with RNF19A expression plasmid or the RNF19A siRNA along with HA‐ubiquitin (Ub). Levels of p53 ubiquitination were detected via immunoprecipitation using the anti‐p53 antibody, followed by anti‐HA immunoblotting

The above results suggested that *RNF19A* knockdown and overexpression increased and decreased p53 expression at the protein level, respectively, while *p53* expression did not change, indicating that RNF19A might regulate p53 at the post‐transcriptional level. As RNF19A is an E3 ubiquitin ligase that can achieve ubiquitination independently, we speculated that RNF19A might interact with p53 and participate in its ubiquitination and degradation. To confirm this hypothesis, *RNF19A*‐silenced A549 and H460 cells were treated with the proteasome inhibitor MG132, and p53 expression was detected via Western blotting. In line with our hypothesis, MG132 rescued the elevated expression of p53 due to *RNF19A* knockdown (Figure 5A). This suggested that RNF19A promoted p53 degradation via the proteasome pathway. We next examined the half‐life of endogenous p53 in A549 and H460 cells which were treated with CHX after RNF19A siRNA transfection. We observed that endogenous p53 in *RNF19A*‐knockdown cells had a longer half‐life than in the scramble siRNA‐treated group (Figure 5B).

We then performed endogenous co‐immunoprecipitation in A549 cells to determine whether RNF19A interacts with p53. The results showed that precipitated p53 immunocomplexes included RNF19A (Figure 5C).

Next, plasmids carrying exogenous FLAG‐*RNF19A* and *p53* were co‐transfected into A549 cells; then, FLAG and p53 were immunoprecipitated, respectively. We found that exogenous RNF19A and p53 co‐immunoprecipitated in both assays (Figure 5D). To further assess whether RNF19A regulates p53 ubiquitination, *RNF19A* was overexpressed or knocked down in A549 cells that were pre‐treated with MG132 to block proteasome‐dependent degradation. We observed that *RNF19A* overexpression distinctly induced p53 ubiquitination, while *RNF19A* knockdown reduced p53 ubiquitination (Figure 5E).

## 4 DISCUSSION

In this paper, we have identified RNF19A as a novel onco‐driver in NSCLC based on two observations: (1) RNF19A was highly expressed in NSCLC tissues, and its overexpression was positively correlated with poor patient outcome in NSCLC; (2) RNF19A increased NSCLC cell proliferation and survival. These results suggested that RNF19A promoted NSCLC development. Previous work has confirmed that several RBR E3 ligases are involved in the regulation of tumour progression by modulating the degradation of tumour promoters or suppressors.^24^ However, the expression pattern, functional implication and prognostic value of RNF19A in NSCLC have been poorly defined.

The canonical homo‐tetrameric p53a protein, also known as p53 or the ‘Guardian of the genome’,^25^ is a powerful and well‐known tumour suppressor, encoded by *TP53*. p53 is a vital transcription factor that plays a crucial role in several cell cycle regulation pathways and induces apoptosis when necessary.^26^ The loss of p53 usually leads to tumorigenesis^27^, ^28^, ^29^ and promotes the occurrence and development of tumours.^30^, ^31^ In this study, we showed that RNF19A not only decreased p53 expression at the protein level but also regulated the downstream signalling of p53, suggesting that RNF19A might promote NSCLC development by decreasing p53 function. The investigation of the potential mechanism indicated that RNF19A (1) promoted proliferation and decreased apoptosis by down‐regulating p53 and (2) regulated p53 downstream signalling, including p21, Cyclin D1, CDK4, CDK6, BAX and BCL2. *p21* was the first gene to be identified to be induced by the wild‐type p53; this induction arrests the cell cycle progression at the G1/S transition through the inhibition of CDK4 and CDK6/cyclin‐D complexes.^32^, ^33^ Cyclin D1, a growth factor sensor, activates CDK4 and CDK6 at the G1 phase.^34^ In addition, p53 triggers a complex network of signals that work through the extrinsic and intrinsic apoptotic pathways. The intrinsic pathway (also called the mitochondrial pathway) promotes the expression of the BCL2 family of proteins towards the pro‐apoptotic members. Under certain conditions, p53 can promote apoptosis by regulating the transcription of particular apoptotic target genes which mediate the majority of apoptotic effects. BAX was the first confirmed p53‐induced member of the BCL2 family, which activates the intrinsic apoptotic pathway by inducing the expression of more than three kinds of BCL2 pro‐apoptotic family members.^35^ In our study, RNF19A decreased p53 expression and modulated the expression of its downstream signalling proteins (p21, BAX, Cyclin D1, CDK4, CDK6 and BCL2). It is worth mentioning that *p53* knockdown significantly, although only partially, rescued the inhibitory effect of *RNF19A* knockdown on the growth of NSCLC cells. Thus, our results strongly indicated that the biological functions of RNF19A might be, at least partially, mediated by the p53 pathway. In contrast, no change in the expression levels of *TP53* was detected, suggesting post‐transcriptional regulation of p53 by RNF19A.

Transcriptional activation of p53 can be mediated by extensive post‐translational modifications including ubiquitination, phosphorylation, acetylation and methylation.^36^, ^37^, ^38^ Ubiquitination is critical for maintaining p53 stability as well as transcriptional regulation of its downstream targets.^39^ It should be noted that p53 is targeted for ubiquitination and degradation by several E3 ligases, including RBR proteins, such as Parkin, RBCK1, RNF31, and CUL9.^40^, ^41^, ^42^, ^43^, ^44^ Given the fact that RNF19A is an RBR E3 ubiquitin ligase that can perform ubiquitination independently, we have been suggested that RNF19A may play a pro‐oncogenic role by regulating p53 ubiquitination. In the present study, consistent with our hypothesis, we found that RNF19A did not only negatively regulate p53 half‐life but also interacted with p53 and induced its ubiquitination. Our study has thus added RNF19A to the already existing list of RBR E3 ubiquitin ligases involved in p53 ubiquitination.

Our study also has some limitations. First, we were unable to analyse the prognosis of our collection of specimens, because the original specimens were collected between 2014 and 2016, and not enough time had passed to collect information. Therefore, prognostic information could only be downloaded from biological databases for further analysis. Second, we have evidence that p53 is at least partially involved in lung tumorigenesis by RNF19A; however, we should not rule out other targets of RNF19A in this complex biological process.

In conclusion, our study has revealed the clinical significance and biological function of RNF19A in NSCLC. RNF19A decreases p53 expression and its downstream signalling, binds to p53 and promotes its ubiquitination, thereby promoting NSCLC growth and progression. RNF19A may thus act as a new biomarker and target for NSCLC prognosis and therapy.

## CONFLICT OF INTEREST

The authors confirm that there are no conflicts of interest.

## AUTHOR CONTRIBUTION

**Yu Cheng:** Conceptualization (lead); Writing‐original draft (lead). **Yujiao Hu:** Methodology (equal). **Huanxi Wang:** Data curation (equal); Methodology (equal). **Zhi Zhao:** Data curation (equal); Methodology (equal); Software (equal). **Xizi Jiang:** Data curation (equal); Methodology (equal); Software (equal). **Yao Zhang:** Data curation (equal); Methodology (equal); Software (equal). **Jiameng Zhang:** Methodology (equal); Resources (equal). **Yue Tong:** Methodology (equal); Resources (equal). **Xueshan Qiu:** Funding acquisition (lead); Project administration (lead); Supervision (lead).

## Supporting information

Fig S1

## Data Availability

The data that support the findings of this study are available from the corresponding author upon reasonable request.
