# Supplementary material for: Ring finger protein 19A is overexpressed in non‐small cell lung cancer and mediates p53 ubiquitin‐degradation to promote cancer growth
Source: J Cell Mol Med. 2021 Jun 29;25(16):7796–808. doi: 10.1111/jcmm.16674 (PMC8358885; doi:10.1111/jcmm.16674)
Supplement: Supplementary file 1 — Fig S1 [file JCMM-25-7796-s001.docx]

**Supporting information**


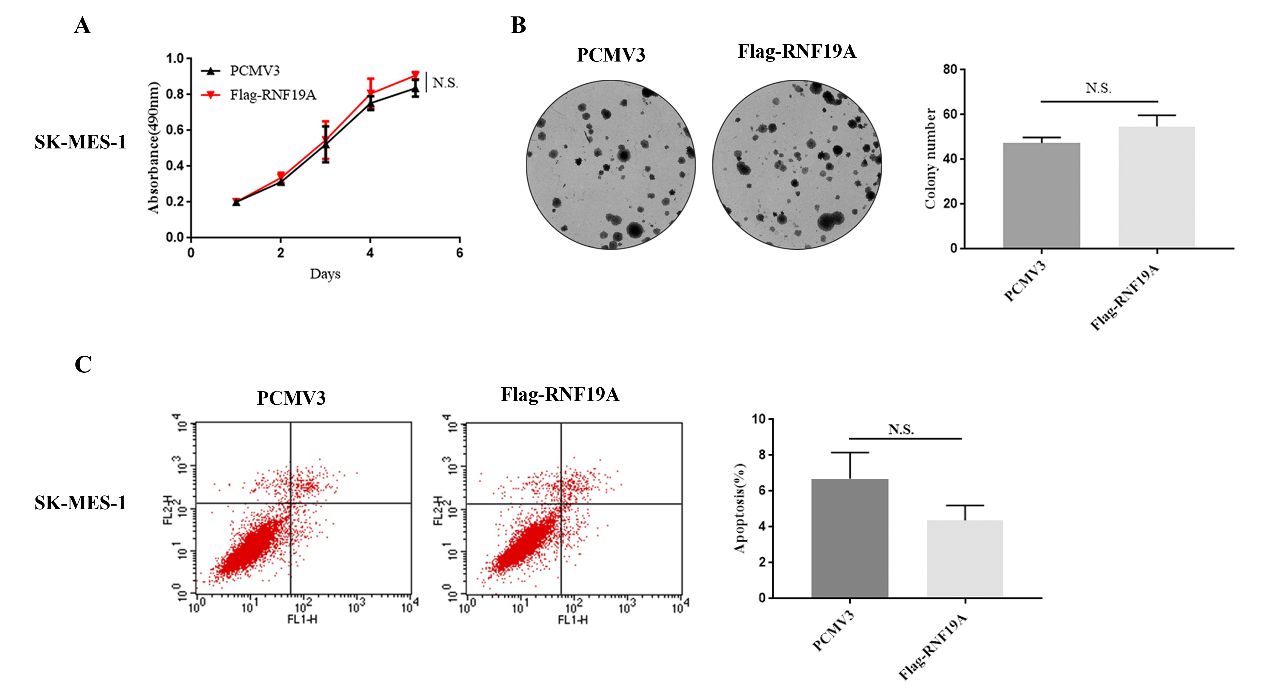


FIGURE1 Effects of overexpression of RNF19A on proliferation and apoptosis of *p53*-mutant (SK-MES-1) cells

1. Overexpression of RNF19A gene had no notable effect on the cell viability of *p53*-mutant (SK-MES-1) cells.
2. Overexpression of RNF19A gene had no notable effect on the clone formation of *p53*-mutant (SK-MES-1) cells.
3. Overexpression of RNF19A gene had no notable effect on the apoptosis of *p53*-mutant (SK-MES-1) cells. Statistical results are presented as the mean ± SE, N.S., not significant.
